# Unilateral Nonconfluent Cluster of Micronodules: Atypical Radiologic Appearance of Pulmonary Tuberculosis in an Immunocompetent Patient

**DOI:** 10.1155/2020/3708252

**Published:** 2020-06-30

**Authors:** Bushra Johari, Mohammad Hanafiah

**Affiliations:** ^1^Department of Radiology, Faculty of Medicine, Universiti Teknologi MARA, UiTM Sungai Buloh Campus, Sungai Buloh 47000, Selangor, Malaysia; ^2^Department of Radiology, Assunta Hospital, Petaling Jaya 46050, Selangor, Malaysia

## Abstract

Active pulmonary tuberculosis involving the lung parenchyma is typically seen on CT as consolidation, centrilobular nodules with tree-in-bud branching, cavitating lesions, and miliary nodules. However, some atypical CT patterns of granulomatous disease including tuberculosis have been recently described, namely, clusters of nodules without confluence or with confluence. We present a case of a patient who was found to have nonconfluent clusters of micronodules in the right lung with negative sputum culture for tuberculosis. There were also incidental findings of the partial duplex system of the left kidney with mild-to-moderate hydronephrosis in the lower moiety with proximal hydroureter. The urine culture was then positive for mycobacterium tuberculosis; hence, he was commenced on antituberculous medications. A repeated CT scan revealed significant improvement of the aforementioned clusters of micronodules and left hydronephrosis. In the present case, we would like to highlight the atypical appearances of pulmonary tuberculosis in the form of nonconfluent micronodules on HRCT despite negative sputum workup, with the concurrent active genitourinary tuberculosis.

## 1. Introduction

On high-resolution computed tomography (HRCT), a lung nodule is defined as well-defined, discrete opacity in the lung parenchyma measuring less than 30 mm. On the other hand, the term micronodule is used to describe a very small nodule. Although a variety of diameters have been used in the past to define a micronodule, it is recommended that the term be reserved for opacity less than 3 mm in diameter [1]. In general, diffuse nodules or nodular patterns are usually described according to their location in the secondary pulmonary lobules, namely, centrilobular, perilymphatic, or random in distribution, as the distribution may give some diagnostic clues to the underlying pathogenesis of the disease.

There are other morphological appearances of nodular patterns such as the “Galaxy Sign” that has been described to be characteristic of granulomatous disease[2]. We, on the other hand, report a patient with a rare and atypical appearance of pulmonary tuberculosis in the form of nonconfluent micronodules on HRCT and highlight the importance of recognizing such morphological nodular pattern.

## 2. Case Presentation

A 65-year-old immunocompetent man presented to the respiratory clinic with recurrent chronic productive cough and gradual loss of weight for 2 years. He denied any fever, haemoptysis, urinary symptoms, or contact to any patients with pulmonary tuberculosis. His initial vital signs were unremarkable (blood pressure = 130/70 mmHg, pulse rate = 76 beats per minute, oxygen saturation = 98% at room air, and temperature = 36.5°C). At the first encounter, he was underweight with a BMI of 17 kg/m^2^. On examination, there was reduced air entry in the left upper lung. The abdomen was soft and nontender. His sputum sample was sent for tuberculosis workup; however, the results were negative. Routine blood investigations including full blood count and urine profile were within normal limits. The inflammatory makers were not elevated with a white cell count of 11 g/dL. His past medical history included benign prostatic hyperplasia and remote history of healed scrotal abscess.

**Figure 1:**
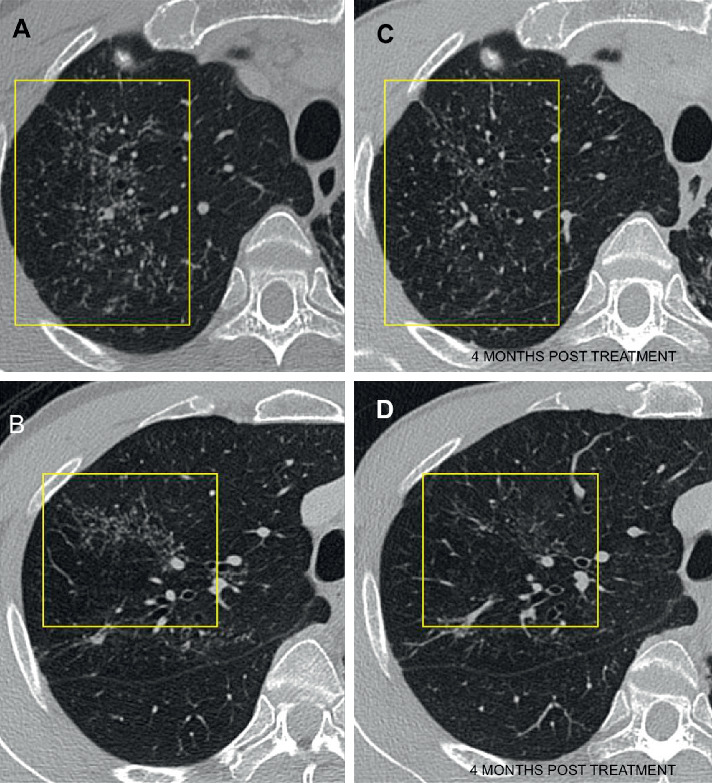
Selected computed tomography (CT) images on axial plane demonstrating clusters of micronodules with no confluence in the right upper lobe (a, b). A repeat CT (c, d) at 4 months after treatment shows improvement of the changes indicating response to anti-TB treatment.

**Figure 2:**
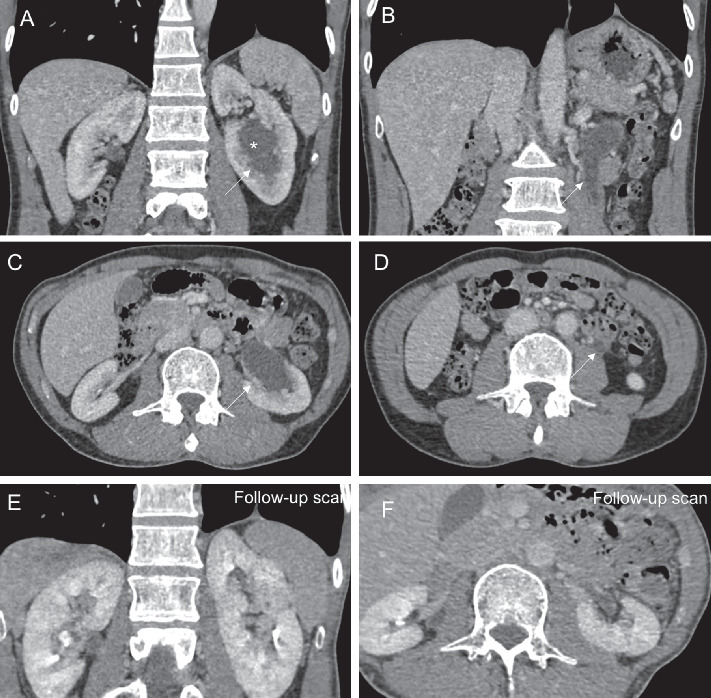
Selected contrast-enhanced CT images of the upper abdomen in coronal and axial planes showing initial (a–d) and posttreatment follow-up (e, f) scans. The images of the initial scan show moderate hydronephrosis and hydroureter (thin white arrows) of the left lower moiety system (^*∗*^), which are largely resolved in the follow-up scan. There were no calculi, ureteric mass, or abdominal lymphadenopathy.

The initial chest radiograph revealed fibrotic lung changes in the upper zones, more so on the left than the right. Subsequently, a contrast-enhanced computed tomography (CT) of the thorax with high-resolution CT reconstruction was performed and showed extensive scarring in both apical regions, again more so on the left. In addition, there were nonconfluent clusters of micronodules (Figure 1) in the right lung with a few smaller regions of tree-in-bud nodularity. There was no significant mediastinal or hilar lymphadenopathy. In the upper part of the abdomen included in the CT, there were also incidental findings of the partial duplex system of the left kidney with mild-to-moderate hydronephrosis in the lower moiety with proximal hydroureter (Figure 2). To further investigate this urinary finding, a multiphasic renal CT scan was then performed and did not reveal any calculus, suspicious ureteric mass or external compression from lymphadenopathy. The culture of his urine sample grew mycobacterium tuberculosis (MTB) complex, and he was diagnosed with active genitourinary and pulmonary tuberculosis. He was commenced on antituberculous medication (Akurit-4). His symptoms improved, and he started to gain weight. A repeated CT scan at 4 months revealed significant improvement of the aforementioned clusters of micronodules, tree-in-bud changes, and left hydronephrosis. Given the positive urine culture for tuberculosis with concurrent respiratory and constitutional symptoms, and improvement of the lung changes and respiratory symptoms following antituberculous treatment, the above-described nonconfluent clusters of micronodules were likely to represent active pulmonary tuberculosis.

## 3. Discussion

Active pulmonary tuberculosis involving the lung parenchyma is typically seen on CT scan as consolidation, centrilobular nodules with tree-in-bud branching, cavitating lesions, and miliary nodules [3]. Some atypical patterns of lung nodules in granulomatous diseases, namely, sarcoidosis and tuberculosis, have been described in the recent literature [2]. The term “Sarcoid Galaxy Sign” was used to describe large parenchymal nodules in sarcoidosis, which are coalescence of multiple tiny nodules with relatively distinct margin of each constituent small nodule [4]. On the other hand, “Sarcoid Cluster Sign,” which is first described by Ortega et al. [5] also in sarcoidosis, refers to clusters of multiple small nodules in the lung parenchyma that are close to each other but are nonconfluent. Similar findings were subsequently reported in emerging papers prompting that these findings are not entirely exclusive to sarcoidosis, rather it can also occur in cases of tuberculosis [6–9]. Hence, the respective terms of “cluster of nodules with confluence” and “cluster of nodules without confluence” were proposed to replace the initial aforementioned signs [10].

Heo et al. [7] also suggested that “clusters of nodules” be taken as a sign of active tuberculosis even in the absence of other typical features, as all the nodules showed improvement with antituberculous medication despite negative sputum examination for acid-fast bacilli in his patients. In these instances, absence of lymphadenopathy and perilymphatic nodules favours tuberculosis compared to sarcoidosis as the underlying pathology [2]. Another sign which has been described as highly suggestive of active granulomatous process, namely, sarcoidosis and tuberculosis, is the “nodular-reversed halo sign.” The term nodular-reversed halo is used to describe a ring of nodules surrounding an area of ground-glass attenuation [10]. This should be differentiated from the more widely described “reversed halo sign,” an area of ground glass opacity surrounded by a ring of consolidation, which can be due to many nonspecific causes including organizing pneumonia and fungal infections among others [11].

In the present case, the unilateral cluster of micronodules without confluence showed significant improvement after antituberculous treatment institution following positive MTB culture in his urine, indicating that MTB was indeed the culprit of this lung changes despite negative sputum workup for pulmonary tuberculosis.

## 4. Conclusion

Cluster of nodules without confluence is not typically seen on HRCT of active pulmonary tuberculosis. However, when encountered, it is quite specific for granulomatous conditions and may be the sole indicator of active disease. Awareness of the existence of this rare atypical radiological manifestation of pulmonary tuberculosis on HRCT is utmost of importance for timely institution of treatment.
